# Yield of array‐CGH analysis in Tunisian children with autism spectrum disorder

**DOI:** 10.1002/mgg3.1939

**Published:** 2022-06-27

**Authors:** Fethia Chehbani, Pasquale Tomaiuolo, Chiara Picinelli, Marco Baccarin, Paola Castronovo, Maria Luisa Scattoni, Naoufel Gaddour, Antonio M. Persico

**Affiliations:** ^1^ Department of Psychiatry, Research Laboratory “Vulnerability to Psychotic Disorders LR 05 ES 10” Monastir University Hospital Monastir Tunisia; ^2^ Faculty of Pharmacy University of Monastir Monastir Tunisia; ^3^ Mafalda Luce Center for Pervasive Developmental Disorders Milan Italy; ^4^ Department of Genetics Synlab Suisse SA Bioggio Switzerland; ^5^ Research Coordination and Support Service Istituto Superiore di Sanità Rome Italy; ^6^ Unit of Child Psychiatry Monastir University Hospital Monastir Tunisia; ^7^ Child & Adolescent Neuropsychiatry Program Modena University Hospital & Department of Biomedical, Metabolic and Neural Sciences, University of Modena and Reggio Emilia Modena Italy

**Keywords:** array CGH, autism spectrum disorder, neurodevelopment, synapse, Tunisia

## Abstract

**Background:**

Autism spectrum disorder (ASD) is a neurodevelopmental disorder with strong genetic underpinnings. Microarray‐based comparative genomic hybridization (aCGH) technology has been proposed as a first‐level test in the genetic diagnosis of ASD and of neurodevelopmental disorders in general.

**Methods:**

We performed aCGH on 98 Tunisian children (83 boys and 15 girls) diagnosed with ASD according to DSM‐IV criteria.

**Results:**

“Pathogenic” or “likely pathogenic” copy number variants (CNVs) were detected in 11 (11.2%) patients, CNVs of “uncertain clinical significance” in 26 (26.5%), “likely benign” or “benign” CNVs were found in 37 (37.8%) and 24 (24.5%) patients, respectively. Gene set enrichment analysis involving genes spanning rare “pathogenic,” “likely pathogenic,” or “uncertain clinical significance” CNVs, as well as SFARI database “autism genes” in common CNVs, detected eight neuronal Gene Ontology classes among the top 10 most significant, including synapse, neuron differentiation, synaptic signaling, neurogenesis, and others. Similar results were obtained performing g: Profiler analysis. Neither transcriptional regulation nor immune pathways reached significance.

**Conclusions:**

aCGH confirms its sizable diagnostic yield in a novel sample of autistic children from North Africa. Recruitment of additional families is under way, to verify whether genetic contributions to ASD in the Tunisian population, differently from other ethnic groups, may involve primarily neuronal genes, more than transcriptional regulation and immune‐related pathways.

## Graphical abstract

Array‐CGH confirms its satisfactory diagnostic yield in a novel sample of 98 Tunisian autistic children. Pathogenic and likely pathogenic CNVs were detected in 11/98 (11.2%) patients. Several recurrent CNVs were found also in this patient population. Preliminary gene pathway analyses suggest that in the Tunisian population, compared to Caucasian and Asian populations, neuronal gene pathways may be more involved with ASD as compared to genes related to transcriptional regulation and immune function. Addditional families are being recruited to verify this hypothesis.

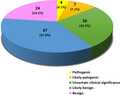

## 1 INTRODUCTION

Autism spectrum disorder (ASD) encompasses a group of neurodevelopmental disorders (the “autisms”) characterized by impairment in social interaction and communication, including deficits in social reciprocity, accompanied by restricted interests, repetitive patterns of behavior, and abnormal sensory processing (American Psychiatric Association, 1994; Persico et al., 2020). Its prevalence rates range from 1/54 children and 1/45 adults in the United States (Dietz et al., 2020; Maenner et al., 2020), to 1/87 children in Italy and 1/102 adults in England (Brugha et al., 2011; Narzisi et al., 2018). ASD has strong genetic underpinnings. Familial recurrence rates are approximately 15%–25% for male and 5%–15% for female offspring (Ozonoff et al., 2011; Persico et al., 2020), much higher than population rates (Brugha et al., 2011; Dietz et al., 2020; Maenner et al., 2020; Narzisi et al., 2018). Twin studies have revealed that heritability can range from 38% to 90%, with approximately 50% as the most likely estimate (Huguet et al., 2016). Genetic contributions to ASD are extremely heterogeneous. The majority of ASD cases fall within a complex genetic‐epigenetic susceptibility model, encompassing a combination of genetic vulnerability conferred by rare and common variants, paired with environmental modulators affecting neurodevelopment through epistatic gene–gene and gene–environment interactions (Bhandari et al., 2020; Chaste et al., 2017; Huguet et al., 2016; Persico & Napolioni, 2013). Traditional cytogenetics, carried out by karyotype on cells in metaphase with the G‐banding method, while very useful in identifying numerical and large structural chromosomal anomalies, has a limited resolution, reaching approximately 3–5 Mb at most. Array‐based technology has produced a dramatic 50‐ to 1000‐fold increase in the detection sensitivity of chromosomal anomalies. Depending on array model and platform, array comparative genomic hybridization (aCGH) allows identifying the presence of CNVs (deletions and duplications) at the whole genome level with a resolution down to just ~5–100 Kb. Hence aCGH, as already extensively described in the literature, has been confirmed as a “gold standard” first‐level test in the genetic diagnosis of ASD (Aradhya et al., 2007; Miller et al., 2010; Sanders et al., 2015) and for neurodevelopmental disorders in general (Baccarin et al., 2020). Much evidence revealed that copy number variants (CNVs) detected by aCGH are major contributors to ASD pathogenesis in up to 10%–15% of affected children (Miller et al., 2010; Sebat et al., 2007). In this study, we have for the first time performed whole‐genome aCGH analysis on a sample of children with ASD recruited in Tunisia.

## 2 PATIENTS AND METHODS

### 2.1 Patients

We enrolled 98 children and adolescents with ASD, including 83 boys and 15 girls (M:F = 5.5:1), ranging from 3 to 18 years old, and belonging to 91 simplex and four multiplex families with two autistic children (both children from each multiplex family were genotyped, except for one family where only one child was genotyped, yielding 91 patients from simplex and seven patients from multiplex families). Patients were recruited between January and April 2017 at the Child and Adolescent Psychiatry Clinic of the Department of Psychiatry, Fattouma Bourguiba University Hospital, Monastir, Tunisia. Clinical diagnosis of ASD was based on the Diagnostic and Statistical Manual of Mental Disorders, Fourth Edition (DSM‐IV) (American Psychiatric Association, 1994) criteria, confirmed by the Autism Diagnostic Inventory‐Revised (ADI‐R) (Lord et al., 1994), and the Autism Diagnostic Observation Schedule‐2 (ADOS‐2) (Lord & Rutter, 2012). The severity of autism was assessed using the Childhood Autism Rating Scales (CARS) (Schopler et al., 1986). All patients had normal karyotype. Patients with fragile‐X syndrome were excluded. This study was approved by the Ethics Committee of Mahdia's University Hospital, Tunisia (Ref. n. *PO9PSY‐2018*). The purpose and protocol of the study were explained to parents by the child psychiatrist (N.G.) and the first investigator (F.C.). Written informed consent was obtained from parents of all patients.

### 2.2 Methods

Peripheral blood was drawn into EDTA‐containing tubes from all probands. Genomic DNA extraction was performed by the salting‐out method (Lahiri & Nurnberger Jr., 1991). aCGH was performed in all patients using the Human Genome CGH SurePrint G3 Microarray 4 × 180 K Kit (Agilent Technologies, Santa Clara, CA), consisting of ∼170.000 60‐mer oligonucleotide probes which span the whole genome with an average spatial resolution of ∼50 Kb. Following the manufacturer's instructions, 200 ng aliquots of genomic DNA from the test and the sex‐matched reference samples were digested with AluI and RsaI (restrictions enzymes). DNA aliquots were labeled with fluorescent nucleotides (Cy3 and Cy5, respectively) and hybridized for 24 hours with an equivalent amount of Cy3 and Cy5 labeled DNA into the microarrays. Slides were finally washed according to manufacturer's instructions and scanned immediately using the DNA Microarray Scanner (Agilent). CNV call was performed using the ADM‐2 algorithm, as implemented in the Agilent Cytogenomic Software v.4.0.3.12 and considering aberrations with at least three consecutive probes. All calls were visually inspected to remove possible false‐positives characterized by irregular Log2 Ratios. In order to ensure reliability, CNVs were defined applying the following parameters: minimum number of probes = 3; if 0 = 2 alleles, mean deletions log_2_ ratio <−0.60 and mean duplication log_2_ ratio >+0.54.

### 2.3 CNVs interpretation

In reference to their frequency in the general population, CNVs were classified into “rare” or “common” using an R script developed ad hoc, based on the presence of ≤3 or >3 healthy subjects, respectively, in the Database of Genomic Variants (DGV, http://projects.tcag.ca/variation/).

In reference to their frequency in the patient population, CNVs were defined as “recurrent” if either identical (i.e., same breakpoints) or similar (i.e., large overlap with <20% difference) to pathogenic/likely pathogenic CNVs carried by patients listed in the DECIPHER database (Firth et al., 2009).

In reference to their clinical significance, CNVs were classified into five categories: (a) pathogenic, (b) likely pathogenic, (c) uncertain clinical significance, (d) likely benign, and (e) benign, in accordance with the 2020 American College of Medical Genetics and Genomics (ACMG) and Clinical Genome Resource (ClinGen) recommendations (Riggs et al., 2020). A two‐step approach was applied: CNVs were first independently classified by three raters (FC, PT, and AMP). The vast majority of first‐pass ratings were convergent, while differences were subject to discussion until full consensus was reached. Subsequently, an additional round of analysis was run by a fourth independent rater (MB) using the following softwares: https://cnvcalc.clinicalgenome.org/cnvcalc/, https://phoenix.bgi.com/autocnv/, and http://autopvs1.genetics.bgi.com/ (Abou Tayoun et al., 2018; Riggs et al., 2020; Xiang et al., 2020). Few differences with scores from the first round were detected, further discussed and finalized. Each patient was finally allocated into one of the five categories, based of the most pathogenic CNV detected in his/her genome. To determine the functional relevance of the CNVs, all information about the genes spanned by each CNV were searched on open‐access databases, including Database of Chromosomal Imbalance and Phenotype in Human using Ensembl Resources (DECIPHER: https://decipher. sanger.ac.uk/application/) (Firth et al., 2009), International Standards for Cytogenomic Arrays Consortium Database (https://isca.genetics.emory.edu), Simons Foundation Autism Research Initiative Gene (SFARI GENE, https://sfari.org/) (Abrahams et al., 2013), and the AutismKB 2.0 database (Yang et al., 2018). Genomic information regarding the chromosomal region duplicated or deleted in each CNV was obtained by searching in the University of California Santa Cruz Genome Browser (http://genome.ucsc.edu), Pubmed, Online Mendelian Inheritance in Man (http://www.ncbi.nlm.nih.gov/Omim), and GeneCards (http://www.genecards.org/). All chromosome coordinates refer to hg19 /GRCh37.

### 2.4 Gene set enrichment analysis and gene ontology

We selected all genes spanning rare CNVs classified as either “pathogenic,” “likely pathogenic,” or “uncertain clinical significance,” as well as all the “autism genes” spanning common CNVs and listed in the SFARI GENE database. We used the open‐access web platform Gene Set Enrichment Analysis (GSEA) (http://software.broadinstitute.org/gsea/index.jsp) to perform Enrichment Analysis with the Gene Ontology Functional database (Subramanian et al., 2005). The FDR method was used to select the top 10 most significant categories, setting statistical significance at p < 0.05 applying a hypergeometric distribution, and exploring dataset C5 from the Molecular Signature Database v7.2 (https://www.gsea‐msigdb.org/gsea/msigdb/).

We performed also a Gene Ontology analysis using g:Profiler (https://biit.cs.ut.ee/gprofiler/gost), a public web server for characterizing and manipulating gene lists (Raudvere et al., 2019). The core of the g:Profiler is g:GOSt, which performs functional enrichment analysis or GSEA, on the input gene list. It maps genes to known functional information sources and detects significantly enriched terms.

## 3 RESULTS

### 3.1 Yield of aCGH analysis

The demographic and clinical characteristics of the sample are presented in Table 1. The majority of children were severely autistic and non‐verbal. All families were simplex, except for four, and 22 (22.5%) children were from consanguineous marriages.

**TABLE 1:** Demographic and clinical characteristic of Tunisian children with ASD (*N* = 98)

Characteristics	N (%)
Sex	
Male	83 (84.7%)
Female	15 (15.3%)
M:F	5.5: 1
Mean age ± SD in years (range)	7.43 ± 3.07 (3–18)
Family type	
Simplex	91
Multiplex (2 ASD siblings)	4^a^
Consanguineous	22 (22.5%)
Language (ADI‐R)	
Verbal	42 (42.9%)
Non‐verbal	56 (57.1%)
CARS	
Mild to moderate ASD	34 (34.7%)
Severe ASD	64 (65.3%)

^a^ Both children were genotyped in three families, only one child was genotyped in one family (total *N* = 7 patients from multiplex families).

**FIGURE 1:**
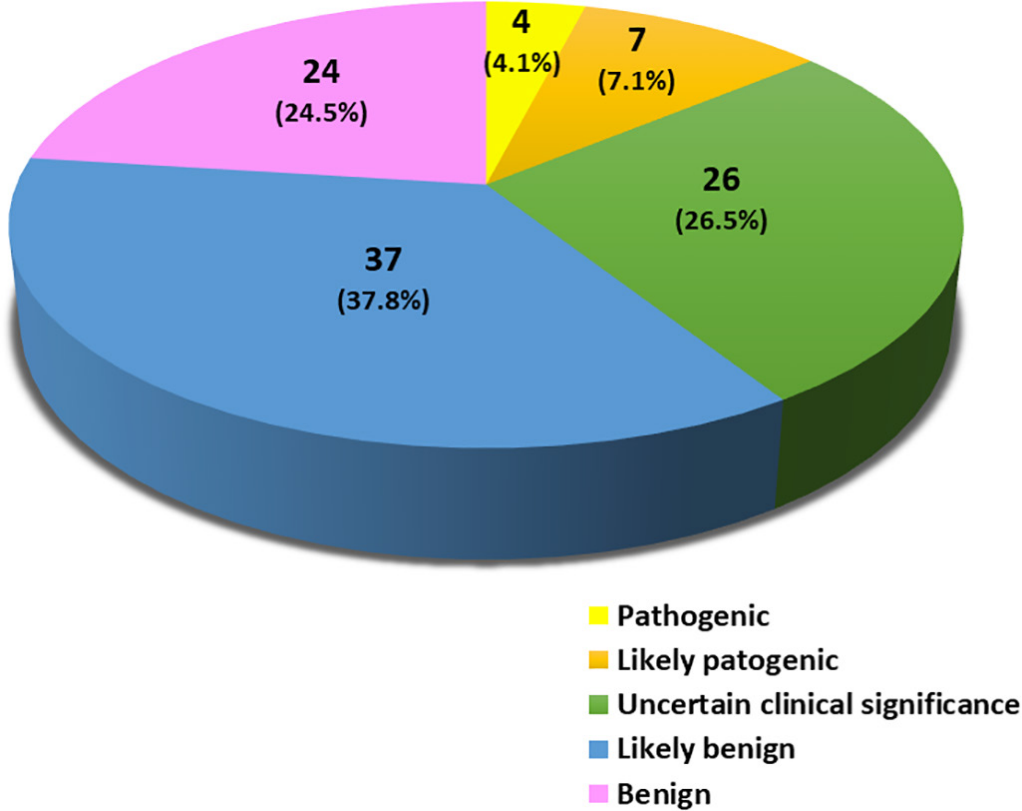
Diagnostic yield of aCGH in Tunisian children with ASD (*N* = 98)

The yield of aCGH analysis is shown in Figure 1. A complete list of all CNVs detected by aCGH analysis is provided as Table S1. Collectively 11 (11.2%) patients, including three females and eight males, carry a total of 14 “pathogenic” or “likely pathogenic” CNVs, encompassing 10 duplications and four deletions (Table 2 and Table S1). Mean size for duplications and deletions is 212.3 kb and 657.6 kb, respectively, with CNVs ranging from 28.7 Kb (chr. 12q13.12 duplication in case n. 12), to over 1.858 Mb (chr. 15q13.2‐q13.3 deletion in case n. 24) (Table 2). Rare CNVs of “uncertain clinical significance” were detected in 26 (26.5%) patients (Table 3). “Likely benign” and “benign” CNVs were detected in 37 (37.8%) patients and 24 (24.5%) patients, respectively (Figure 1).

**TABLE 2:** Rare CNVs defined “pathogenic” or “likely pathogenic” based on ACMG criteria (Riggs et al., 2020), detected in 98 Tunisian children with ASD

Id. n.	Sex	Chr	Band	Start	End	Length	Dup/Del	Genes (*OMIM n.)	Pathogenic/likely pathogenic	Clinical phenotype	Identical CNV on Decipher	Similar CNV on Decipher
12	M	6	q26	162,847,908	163,036,272	188,364	Del	**PRKN** (* 602544)	Likely pathogenic	ASD	No	Yes
23	M	15	q13.2 ‐ q13.3	30,652,489	32,510,863	1,858,374	Del	CHRFAM7A (* 609756), GOLGA8R, ULK4P1, ULK4P2, GOLGA8H, **ARHGAP11B** (* 616310), LOC100288637, HERC2P10, **FAN1** (* 613534), MTMR10 (% 208500), **TRPM1** (* 603576), MIR211 (* 613753), LOC283710, KLF13 (* 605328), **OTUD7A** (* 612024), **CHRNA7** (* 118511)	Pathogenic	ASD	No	Yes
24	F	22	q13.33	50,999,319	51,178,264	178,945	Del	SYCE3 (* 615775), CPT1B (* 601987), CHKB‐CPT1B, BC048192, **CHKB** (* 612395), CHKB‐AS1, MAPK8IP2 (* 607755), ARSA (* 607574), **SHANK3** (* 606230), AC000036.4, ACR (* 102480)	Pathogenic	ASD, ID	No	Yes
33	M	16	p12.2	21,973,762	22,358,382	384,620	Dup	UQCRC2 (* 191329), PDZD9, C16orf52, VWA3A, **EEF2K** (* 606968), POLR3E (* 617815), **CDR2** (* 117340)	Likely pathogenic	ASD, ADHD	No	Yes
41	M	16	p11.2	29,652,999	30,197,341	544,342	Del	SPN (* 182160), QPRT (* 606248), C16orf54, ZG16 (* 617311), KIF22 (* 603213), MAZ (* 600999), PRRT2 (* 614386), PAGR1 (* 612033), MVP (* 605088), CDIPT (* 605893), CDIPT‐AS1, **SEZ6L2** (* 616667), ASPHD1, **KCTD13** (* 608947), TMEM219, **TAOK2** (* 613199), HIRIP3 (* 603365), INO80E, DOC2A (* 604567), C16orf92 (* 618911), FAM57B (* 615175), ALDOA (* 103850), PPP4C (* 602035), TBX6 (* 602427), YPEL3 (* 609724), LOC101928595, GDPD3 (* 616318), MAPK3 (* 601795), CORO1A (* 605000)	Pathogenic	ASD	No	Yes
42	M	X	p21.2	31,069,677	31,382,751	313,074	Dup	FTHL17 (* 300308), **DMD** (* 300377), JA783813,MIR584, RNU6‐8940, JA783807	Pathogenic	ASD	No	No
57	F	X	p22.31	6,081,799	6,445,321	363,522	Dup	**NLGN4X**, MIR4770	Likely pathogenic	ASD, ID, PKU, epilepsy	No	No
83	M	8	p23.3	637,318	1,196,615	559,297	Dup	**ERICH1**, ERICH1‐AS1, LOC401442	Likely pathogenic	ASD	No	No
87	F	X	q13.3	73,950,867	74,152,687	201,820	Dup	**NEXMIF** (* 300524)	Likely pathogenic	ASD, ID	No	No
95	M	18	q12.2	34,828,624	35,158,932	330,308	Dup	**CELF4** (* 612679), LOC105372068, SNORA111	Likely pathogenic	ASD, ID	No	No

*Note*: All genes present in each CNV are listed. OMIM genes are followed by their OMIM id. n. Genes highlighted in bold are most responsible for pathogenicity score.

Abbreviations: ASD, autism spectrum disorder; ID, intellectual disability; PKU, phenylketonuria.

**TABLE 3:** Rare CNVs of “uncertain clinical significance” based on ACMG criteria (Riggs et al., 2020), detected in 98 Tunisian children with ASD

Id. n.	Sex	Chr	Band	Start	End	Length	Dup/Del	Genes (OMIM n.)	Identical CNV on Decipher	Similar CNV on Decipher	Clinical phenotype
2	M	11	p11.2	44,242,500	44,305,707	63,207	Dup	ALX4 (* 605420), **EXT2** (* 608210)	No	No	ASD, regression
3, 4	M	2	q23.3	152,849,387	152,869,902	20,515	Del	**CACNB4** (* 601949)	No	No	ASD
5	F	10	q21.3	68,087,319	68,479,279	391,960	Del	**CTNNA3** (* 607667)	No	No	ASD, ID, epilepsy, regression.
		X	q28	148,736,043	148,798,821	62,778	Dup	**MAGEA11** (* 300344)	No	No	
49	F	X	p22.33	1,737,815	1,783,772	45,957	Dup	**ASMT** (* 300015)	No	Yes	ASD
2, 6, 15, 16, 47, 59, 70, 71		Y	p11.32	1,681,671	1,698,307	16,636	Dup	**ASMT** (* 300015)	No	No	ASD
9	M	3	q11.2	97,875,805	97,981,668	105,863	Dup	OR5H15	No	No	ASD, ID
		4	p16.1	9,558,084	9,803,555	245,471	Del	**DRD5** (* 126453)	No	No	
18	M	5	p14.3	20,642,797	20,712,049	69,252	Del	AK093362, CDH18 is 67 kb away	No	Yes	ASD, ID
		X	p11.21	54,800,624	54,834,642	34,018	Dup	**ITIH6**, MAGED2 (* 300470)	No	No	
21	F	5	p13.1	41,487,476	41,585,063	97,587	Del	PLCXD3 (* 617016)	No	No	ASD
		13	q31.3	94,319,604	94,382,644	63,040	Del	**GPC6** (* 604404)	No	No	
26	M	1	p13.1 ‐ p12	117,597,940	117,980,448	382,508	Dup	LOC101929099, TTF2 (* 604718), MIR942, TRIM45 (* 609318), VTCN1 (* 608162), LINC01525, MAN1A2 (* 604345)	No	No	ASD, ID
		3	p26.1	4,679,246	4,927,297	248,051	Dup	**ITPR1** (* 147265), EGOT	No	No	
33, 37, 69,76		X	q28	154,396,991	154,425,684	28,693	Del	**VBP1**	272,647	Yes	ASD
35	M	4	q25	111,528,886	111,587,488	58,602	Dup	**PITX2** (* 601542)	No	No	ASD
44, 45, 59		14	q22.1	51,080,352	51,156,393	76,041	Dup	ATL1, SAV1	No	Yes	ASD
48	M	12	q14.3	66,877,353	66,901,498	24,145	Del	**GRIP1** (* 604597)	No	No	ASD
		X	q26.3	134,688,525	134,800,326	111,801	Dup	INTS6L	No	No	
61	M	11	q23.3	117,266,818	117,343,844	77,026	Dup	CEP164 (* 614848), **DSCAML1** (* 611782)	No	No	ASD, ID
62	M	12	q13.12	49,359,096	49,387,796	28,700	Dup	WNT10B (* 601906), **WNT1** (* 164820)	No	No	ASD
72	M	19	q11 ‐ q12	28,272,497	29,106,240	833,743	Dup	LINC00662, LOC101927151, LOC100420587	No	Yes	ASD
		20	q12	41,480,071	41,532,148	52,077	Dup	**PTPRT** (* 608712)	No	No	
77	M	14	q23.1	61,067,907	61,203,283	135,376	Dup	SIX1 (* 601205), **SIX4** (* 606342), MNAT1 (* 602659)	No	Yes	ASD
80	M	19	p13.2	7,676,263	7,879,580	203,317	Dup	CAMSAP3 (* 612685), MIR6792, XAB2 (* 610850), PET100 (* 614770), PCP2 (* 619344), STXBP2 (* 601717), RETN (* 605565), MCEMP1 (* 609565), TRAPPC5 (* 614781), FCER2 (* 151445), CLEC4G (* 616256), CD209 (* 604672), CLEC4M (* 605872), CLEC4GP1	No	Yes	ASD, ID, epilepsy, regression
82	M	5	p13.2	37,487,581	37,576,773	89,192	Del	**WDR70** (* 617233)	No	No	ASD, ID
		17	q25.1	71,016,656	71,051,102	34,446	Del	**SLC39A11** (* 616508)	No	No	ASD, ID

*Note*: All genes present in each CNV are listed. OMIM genes are followed by their OMIM id. n. Genes highlighted in bold are most responsible for pathogenicity score.

Abbreviations: ASD, autism spectrum disorder; ID, intellectual disability.

### 3.2 Pathogenic/Likely pathogenic CNVs

At least five individuals carry rare “pathogenic” or “likely pathogenic” dup/dels similar to CNVs listed in the Decipher database, although none is exactly overlapping (Table 2). Three of these recurrent CNVs are certainly pathogenic:

- Patient n.41 is a 6‐year‐old boy carrying a large deletion of >500 kb in size, located in chr. 16p11.2 (“16p11.2 microdeletion syndrome”). This child shows severe deficits in social communication, language delay with echolalia, and other moderate symptoms of ASD, in line with prior clinical descriptions of this syndrome (Marshall & Scherer, 2012). The deletion spans 29 genes, including some exerting very important neurodevelopmental functions (*QPRT, MVP, TAOK2, MAPK3*, and *TBX6*).
- Patient n.23 is a 9‐year‐old nonverbal boy with severe ASD and cognitive impairment, who carries the largest deletion spanning over 1.85 Mb in size, located at chr. 15q13.2‐q13.3 (“15q13.3 microdeletion syndrome”). This region comprises several OMIM genes, including *OTUD7A* and *CHRNA7*. The primary candidate for the phenotypic consequences of 15q13.3 microdeletion syndrome is *OTUD7A* (Forsingdal et al., 2016; Uddin et al., 2018; Yin et al., 2018), with *CHRNA7* likely playing a modulatory role (Bacchelli et al., 2015; Marotta et al., 2020) (see Discussion).
- Patient n.24 is an 11‐year‐old nonverbal girl diagnosed with severe ASD and ID (IQ estimated at 25). She carries a terminal deletion of approximately 178 kb located on chr. 22q13.33, involving the *SHANK3* gene. This deletion causes Phelan‐McDermid syndrome (PMS), a rare genetic disorder mainly characterized by ID, global developmental delay, muscle hypotonia, and motor coordination deficits, accompanied by ASD in approximately 40% of cases, and by several other systemic signs and symptoms (Yi et al., 2016).

Most other “pathogenic” and “likely pathogenic” CNVs do not overlap nor appear similar to CNVs listed in Decipher, but they do span “autism genes” listed in the *SFARI GENE*, and AutismKB databases, such as *DMD, NEXMIF, NLGN4X, PRKN*, and others (Table 2). Their role in ASD and in other neurodevelopmental disorders has been reported in several studies (Aradhya et al., 2007; Bourgeron, 2015; Morato Torres et al., 2020; Satterstrom et al., 2020; Yuen et al., 2017). Patient n. 42 is a 5‐year‐old boy who carries an Xp21.2 duplication involving exons 61–79 of the full‐length isoform and the entire shortest isoform of the dystrophin (*DMD*) gene. *DMD* deletions, duplications, and mutations cause Duchenne muscular dystrophy (DMD), Becker muscular dystrophy (BMD), or cardiomyopathy [OMIM #300377]. This boy was referred for autistic behaviors and has not yet developed a clear DMD/BMD or cardiomyopathy phenotype, as previously observed in other young children (Pagnamenta et al., 2011; Qiao et al., 2013; Watson et al., 2020). Another rare “likely pathogenic” CNV of interest was detected in patient n. 87, a 10‐year‐old nonverbal autistic girl with ID, who carries a duplication at chr. Xq13.3 encompassing the entire Neurite Extension and Migration Factor (*NEXMIF* or *KIAA2022*) gene, while sparing the neighboring *RLIM* gene, which has recently been implicated in a mild neurocognitive phenotype in hemizygous males (Palmer et al., 2020). Patient n. 57 is an 11‐year‐old girl who carries a partial exonic duplication involving the *NLGN4X* gene. She was also diagnosed with phenylketonuria (PKU), which is associated with ASD especially in late‐diagnosed PKU patients, although full comorbidity remains a rare event (Baieli et al., 2003; Bilder et al., 2017). Finally patient n. 12 is a 6‐year‐old boy carrying a deletion involving the 5′ end (i.e., exons 1, 2 and/or 3 depending on the isoform) of the *PRKN* gene, also known as *PARK2*. Mutations and deletions in this gene have been *bona fide* associated with Parkinson's disease, but rare CNVs are also associated with ASD and other neurodevelopmental disorders (Morato Torres et al., 2020).

In addition to rare CNVs listed in Table 2, also common CNVs can confer some degree of liability to ASD (Monteiro et al., 2019; Toma, 2020). In particular, we detected the recurrent 15q11.1‐q11.2 BP1‐BP2 microduplication (Chai et al., 2003), encompassing *NIPA1, NIPA2, CYFIP1*, and *TUBGCP5* in a 13‐year‐old boy with moderate autism (case n. 44 in Table S1). This variant, though not rare, was scored as “likely pathogenic” because epistatic interactions between this CNV and additional rare or common variants can lead to the appearance of distinct behavioral phenotypes (Picinelli et al., 2016), although contrasting interpretations have been presented (Chaste et al., 2014).

### 3.3 Variants of unknown significance (VOUS)

Rare VOUS are listed in Table 3. Duplications slightly exceed the number of deletions (dup/del = 16:14), as reported in another study (Siu et al., 2016), tend to be larger than deletions and on average encompass more genes. Among the 26 patients carrying VOUS, 9 (9.2%) present partial rare duplications in the acetylserotonin O‐methyltransferase (*ASMT*) gene, located in the pseudoautosomal region 1 of the sex chromosomes (Table 3). *ASMT* encodes for the last enzyme of melatonin synthesis, mutations in this gene have been associated with ASD, and low levels of melatonin have been recorded in many autistic children (Ballester et al., 2020; Jonsson et al., 2014; Melke et al., 2008; Wang et al., 2013). In our cohort, 6 (66.7%) out of the 9 patients carrying *ASMT* duplications reported sleep disorders, in contrast to 36 (36.8%) out of the 98 patients in the entire sample, underscoring the importance of CNVs involving *ASMT* in influencing sleep and circadian rhythms.

Three boys (id. n.44, 45, and 59) carry the same 14q22.1 duplication encompassing *ATL1*, one of the causative genes of spastic paraplegia (Table 4). These patients come from the same geographical area of origin (Gafsa) in Tunisia. Cases n. 44 and 45 are first‐degree cousins and display no language impairment, whereas patient n. 65 is a 9‐year‐old unrelated nonverbal boy. *ATL1* encodes a protein involved in the regulation of endoplasmic reticulum (ER) morphology (Shih & Hsueh, 2018). The loss of *ATL1* function leads to abnormalities in axonal development, impairment of neuronal growth (Zhu et al., 2006), and a reduction of dendritic spine density (Shih & Hsueh, 2018). The implication of this rare CNV in the etiology of ASD is still unknown, but contributions to ASD pathogenesis by ER formation in its interaction with the Golgi system cannot be excluded. Similarly, in cases n. 44 and 65 epistatic interactions with CNVs involving *ASMT* and the 15q11.1‐q11.2 BP1‐BP2 microduplication described above also cannot be excluded (Table 3 and Table S1).

**TABLE 4:** Gene Set Enrichment Analysis (GSEA) for gene ontologies of 207 genes spanning CNVs scored as “pathogenic,” “likely pathogenic,” or of “uncertain clinical significance” in Tunisian children with ASD (*N* = 98)

Gene Set Name	Genes	# Genes in Overlap (k)	# Genes in Gene Set (K)	k/K	*p*‐value	FDR *q*‐value
GO_SYNAPSE	SHANK3, PRKN, CHRNA7, CYFIP1, NLGN4X, MAPK8IP2, SYT17, GRIP1, EEF2K, DSCAML1, CNTN6, PRRT2, DRD5, DLGAP2, CHRFAM7A, DOC2A, CACNB4, EPS8, CORO1A, DMD, ITPR1, GPC6	22	1357	1.62	2.04E‐7	1.12E‐3
GO_GOLGI_CIS_CISTERNA	ATL1, GOLGA8DP, GOLGA8R, GOLGA8H, GOLGA8CP	5	30	16.67	2.19E‐7	1.12E‐3
GO_NEURON_DIFFERENTIATION	SHANK3, PRKN, CHRNA7, CYFIP1, NLGN4X, MAPK8IP2, SYT17, GRIP1, EEF2K, DSCAML1, CNTN6, ATL1, SIX1, WNT10B, TAOK2, MAPK3, TRPM1, WNT1, MOSMO, PITX2, TBX6	21	1406	1.49	1.46E‐6	3.13E‐3
GO_SYNAPTIC_SIGNALING	SHANK3, PRKN, CHRNA7, CYFIP1, NLGN4X, MAPK8IP2, SYT17, PRRT2, DRD5, DLGAP2, CHRFAM7A, DOC2A, CACNB4, CELF4, KCTD13	15	751	2.00	1.64E‐6	3.13E‐3
GO_FUNGIFORM_PAPILLA_ DEVELOPMENT	SIX1, WNT10B, SIX4	3	6	50.00	1.74E‐6	3.13E‐3
GO_NEUROGENESIS	SHANK3, PRKN, CHRNA7, CYFIP1, NLGN4X, MAPK8IP2, SYT17, GRIP1, EEF2K, DSCAML1, CNTN6, ATL1, SIX1, WNT10B, TAOK2, MAPK3, TRPM1, WNT1, MOSMO, PITX2, TBX6, SIX4, NEXMIF	23	1674	1.37	1.83E‐6	3.13E‐3
GO_REGULATION_OF_TRANS_ SYNAPTIC_SIGNALING	SHANK3, PRKN, CHRNA7, CYFIP1, NLGN4X, MAPK8IP2, PRRT2, DRD5, DLGAP2, CELF4, KCTD13	11	455	2.42	7.2E‐6	1.06E‐2
GO_SYNAPTIC_GROWTH_AT_ NEUROMUSCULAR_JUNCTION	SHANK3, SIX1, SIX4	3	10	30.00	1.03E‐5	1.32E‐2
GO_NEURON_PROJECTION	SHANK3, PRKN, CHRNA7, CYFIP1, NLGN4X, GRIP1, EEF2K, DSCAML1, CNTN6, PRRT2, CHRFAM7A, DOC2A, EPS8, CORO1A, DMD, ATL1, TAOK2, SAV1, ADAM21	19	1366	1.39	1.3E‐5	1.43E‐2
GO_SIGNALING_ADAPTOR_ ACTIVITY	SHANK3, MAPK8IP2, GRIP1, EPS8, SAV1	5	68	7.35	1.4E‐5	1.43E‐2

Finally, other deletions are located near important causative autism genes, such as *VAMP7* and *CDH18*, and contain highly conserved H3K27Ac marks often found near active regulatory elements (see http://genome.ucsc.edu). In principle, CNVs can influence gene expression by affecting epigenetically relevant regions encompassing long noncoding RNAs (Bilinovich et al., 2019; DeWitt et al., 2016) or able to modulate DNA methylation, histone modifications, and the expression of noncoding micro‐RNAs (Wiśniowiecka‐Kowalnik & Nowakowska, 2019). Until demonstrated epigenetically relevant, these CNVs remain of unknown significance.

### 3.4 Pathway analysis by gene set enrichment analysis and gene ontology

Pathway analysis was conducted using both the GSEA web platform (Subramanian et al., 2005) and g:Profiler (Raudvere et al., 2019), on a total of 207 genes, spanning CNVs scored as “pathogenic,” “likely pathogenic,” or of “uncertain clinical significance.” Results are shown in Tables 4 and 5, respectively. GSEA and g:Profiler confirmed that most gene categories with the highest statistical significance are nervous system‐specific and that many of the genes spanned by these CNVs are already associated with ASD and/or neurodevelopmental disorders. In particular, the complete top 10 most enriched gene sets identified by GSEA are listed in Table 4. Partly overlapping sets of genes identified GO Synapse, Neuron Differentiation, Synaptic Signaling, Neurogenesis, Regulation of Trans‐synaptic Signaling, Synaptic Growth at Neuromuscular Junction, Neuron Projection, and Signaling Adaptor Activity (Table 4). Only two pathways, GO Golgi Cis Cisterna and Fungiform Papilla Development fall outside this framework. Similarly, enrichment analyses performed using g:Profiler identified 11 overrepresented Gene Ontology categories, including five biological processes and six cellular components (Table 5a and b). Among biological process, at least two are neuron‐specific, namely negative regulation of synaptic transmission and modulation of excitatory postsynaptic potential. Instead, all six cellular components regard synaptic structure and function (Table 5b). Single genes, for example *CELF4* (Table 2), indeed play important roles in mRNA processing, but in reference to gene ensembles, neither “transcriptional regulation” nor “immune” pathways reached statistical significance.

**TABLE 5:** g:Profiler analysis of single genes and relative gene ontologies for [a] Biological Processes and [b] Cellular Components

[a] Biological process	GO term	Adjusted *p*‐value	Genes input	Total gene	Genes/total (%)	Genes
Cell–cell recognition	GO:0009988	0.0148	6	79	7.59	DOCK8, ARSA, ACR, ADAM21, ADAM20, ALDOA
Negative regulation of synaptic transmission	GO:0050805	0.0118	6	76	7.89	DRD5, PARK2, CHRFAM7A, SHANK3, NLGN4X, CELF4
Modulation of excitatory postsynaptic potential	GO:0098815	0.0148	5	46	10.87	CHRFAM7A, CHRNA7, SHANK3, NLGN4X, CELF4
Binding of sperm to zona pellucida	GO:0007339	0.0094	5	42	11.90	ARSA, ACR, ADAM21, ADAM20, ALDOA
Fungiform papilla development	GO:0061196	0.0076	3	6	50.00	WNT10B, SIX1, SIX4

[b] Cellular component	GO term	Adjusted *p*‐value	Genes input	Total gene	Genes/total (%)	Genes
Synapse	GO:0045202	0.0058	21	1293	1.62	SYT17, CACNB4, DRD5, PRKN, GPC6, CHRFAM7A, CHRNA7, MAPK8IP2, SHANK3, ITPR1, EEF2K, PRRT2, DOC2A, CORO1A, DMD, CYFIP1, GRIP1, NLGN4X, DSCAML1, EPS8, PTPRT
Postsynapse	GO:0098794	0.0137	13	591	2.20	CHRFAM7A, CHRNA7, MAPK8IP2, SHANK3, ITPR1, EEF2K, PRRT2, DMD, CYFIP1, GRIP1, NLGN4X, EPS8, PTPRT
Neuron to neuron synapse	GO:0098984	0.0075	10	329	3.04	MAPK8IP2, SHANK3, ITPR1, EEF2K, PRRT2, DMD, GRIP1, NLGN4X, EPS8, PTPRT
Postsynaptic specialization	GO:0099572	0.0011	11	328	3.35	CHRNA7, MAPK8IP2, SHANK3, ITPR1, EEF2K, PRRT2, DMD, GRIP1, NLGN4X, EPS8, PTPRT
Asymmetric synapse	GO:0032279	0.0046	10	311	3.22	MAPK8IP2, SHANK3, ITPR1, EEF2K, PRRT2, DMD, GRIP1, NLGN4X, EPS8, PTPRT
Postsynaptic density	GO:0014069	0.0039	10	305	3.28	MAPK8IP2, SHANK3, ITPR1, EEF2K, PRRT2, DMD, GRIP1, NLGN4X, EPS8, PTPRT

## 4 DISCUSSION

Our study replicates and extends previous results underscoring the importance of aCGH for uncovering chromosomal imbalances and genomic rearrangements in individuals with ASD, as well as other neurodevelopmental disorders (Aradhya et al., 2007; Iourov et al., 2012; Miller et al., 2010; Monteiro et al., 2019; Sanders et al., 2015; Siu et al., 2016; Wall et al., 2009). We detected “pathogenic” or “likely pathogenic” CNVs in 11/98 (11.2%) children with ASD, the first recruited and clinically well‐characterized sample from North Africa assessed using this technology. This detection rate largely overlaps with previous findings from groups applying similar technologies (Monteiro et al., 2019; Siu et al., 2016), demonstrating that ethnicity does not significantly influence the relative weight of CNVs in the pathogenesis of ASD. Among children with “pathogenic” and “likely pathogenic” CNVs, duplications were more frequent than deletions. Similar to previous studies, the size of pathogenic CNVs is larger and encompasses more genes compared to VOUS, “likely benign” and “benign” CNVs altogether (Aradhya et al., 2007; Cappuccio et al., 2016). Otherwise, deletions and duplications may differentially affect social communication, behavior, and phonological memory, whereas both equally affect motor skills (Douard et al., 2021).

Several recurrent risk loci carrying increased ASD liability, such as 15q13.3, 16p11.2, and 22q13.33, have been detected in this sample and may be associated with relatively specific phenotypes (Chaste et al., 2017; Huguet et al., 2016):

- Individuals with chromosome 16p11.2 microdeletion syndrome frequently display peculiar clinical features, mostly present also in our case n. 41, including higher rates of developmental abnormalities, lower IQ, birth complications, medical issues, and an equal sex ratio (Marshall & Scherer, 2012). More broadly, both deletions and duplications in the 16p11.2 region enhance the risk for ASD (Chaste et al., 2017), while only duplications are related to schizophrenia (Szatkiewicz et al., 2014).
- Chr. 15q13.2‐q13.3 microdeletion syndrome can result in neurodevelopmental phenotypes as diverse as schizophrenia, epilepsy, and autism (Bacchelli et al., 2015; Ben‐Shachar et al., 2009; Dibbens et al., 2009; Gillentine & Schaaf, 2015). Its critical region comprises several OMIM genes, including *OTUD7A* and *CHRNA7*. *OTUD7A* encodes one of a family of deubiquitinases mainly expressed in the central nervous system (CNS), where it stimulates dendritic growth and spine development, enhancing excitatory synaptic activity (Uddin et al., 2018; Yin et al., 2018). *OTUD7A* ko mice largely recapitulate the clinical signs and symptoms of human patients, underscoring the pivotal role of this gene (Forsingdal et al., 2016; Uddin et al., 2018; Yin et al., 2018). A modulatory effect is instead played by *CHRNA7*, which encodes the alpha 7 subunit of the nicotinic acetylcholine receptor, localized both pre‐ and postsynaptically (Papke & Lindstrom, 2020; Zoli et al., 2018). In addition to its ionotropic neuronal functions, modulating the secretion of both the inhibitory neurotransmitter GABA and the excitatory neurotransmitter glutamate, the α7 nicotinic receptor is also able to regulate neuroinflammation through independent metabotropic effects detected in many nonneuronal cell types (Papke & Lindstrom, 2020; Zoli et al., 2018). The differential burden of *OTUD7A* and *CHRNA7* is well exemplified by a recent report describing a boy and a girl, each carrying two contiguous 15q13.3 microdeletions involving *CHRNA7* and displaying very different clinical phenotypes: the boy has severe cognitive impairment and hyperactivity, while the girl had mild language deficits and a high IQ (Alsagob et al., 2019). Interestingly, the downstream deletion includes both *OTUD7A* and *CHRNA7* in the severely affected boy, while only *CHRNA7* is deleted in the mildly affected girl (Alsagob et al., 2019). In line with this evidence, our case n. 23 carries a deletion involving both genes and is severely affected with ASD, cognitive impairment and lack of verbal language.
- Chr. 22q13.33 terminal deletions result in Phelan‐McDermid syndrome (PMS) which in our case n. 24 displays a particularly severe phenotype. Pathogenic deletions and mutations typically involve the *SHANK3* gene (OMIM #606230), which encodes a scaffolding protein that is enriched in postsynaptic densities of excitatory synapses and also in presynaptic axons and terminals (Wang et al., 2014). In the CNS, SHANK proteins play a role in synapse formation and dendritic spine maturation. The *SHANK3* gene may yield more than a hundred isoforms in the brain, each with a unique function possibly relevant to the diversity and specificity of each type of synapse (Wang et al., 2014). Besides its crucial role in the CNS, *SHANK3* is also involved in the maturation of neuromuscular junctions and in striated muscles. Hence, *SHANK3* haploinsufficiency invariably results in muscle hypotonia, by interfering with each element of the transmission chain involved in voluntary movements, namely motoneurons, neuromuscular junctions, and striated muscles (Lutz et al., 2020; Waga et al., 2011). *SHANK3* deletions and mutations have been detected in ~0.5% of ASD patients (Moessner et al., 2007; Waga et al., 2011; Wang et al., 2014), making this gene a strong monogenic cause of low‐functioning ASD, as in the case of our patient n. 24. Together with *SHANK3*, other genes present in the 22q13.3 region can also contribute to interindividual differences in phenotype (Ricciardello et al., 2021). In particular, the deletion carried by patient n. 24 also encompasses *MAPK8IP2*, a recurrent gene in our GSEA analyses (Tables 4 and 5) which could enhance the severity of autistic features (Ricciardello et al., 2021).

Among other genes playing a “pathogenic” or “likely pathogenic” role, *DMD* is especially interesting, because duplications have been found in children diagnosed primarily with ASD and not with DMD/BMD. This may be due both to its earlier onset and at least in some cases due to a milder expression of the muscular phenotype, which can clinically overlap with the motor development delays and coordination deficits often seen in children with ASD (Pagnamenta et al., 2011; Qiao et al., 2013; Watson et al., 2020). Also the chr. Xq13.3 duplication involving *NEXMIF* is interesting, because it spares the neighboring *RLIM*, recently proposed as the strongest candidate gene for the cognitive and dysmorphological phenotype of these patients (Palmer et al., 2020). Instead our case n. 87 draws attention onto *NEXMIF*, previously known as *KIAA2022*, which is highly expressed in the brain and encodes the X‐linked Intellectual Disability Protein Related to Neurite Extension (XPN) protein, crucial to neuronal migration, cell adhesion, neurite outgrowth, and branching (Hansen et al., 2008). Different types of *NEXMIF* gene imbalances may act as hemizygous or compound heterozygous loss‐of‐function mutations, responsible for a form of X‐linked ID [OMIM 300524]. Patients with *NEXMIF* mutations may share some phenotypic features, such as language delay, refractory seizures, dysmorphism, and autistic traits (Webster et al., 2017). Finally, the partial exonic duplication involving the *NLGN4X* gene found in case n. 57 points toward probable contributions by this CNV to the comorbidity of ASD and PKU in this girl. Indeed there is no evidence of direct links between *NLGN4X* and PKU. However, PKU and ASD, despite being significantly associated in comparison with population prevalence rates, co‐occur only in a small minority of PKU patients (Baieli et al., 2003; Bilder et al., 2017). Hence, this duplication involving *NLGN4X* may likely increase the probability of observing an autistic phenotype in a child with PKU .

GSEA categories “synaptic signaling” and “excitatory postsynaptic potential” are especially enriched in genes spanning “pathogenic” and “likely pathogenic” CNVs (Table 4). Previous studies have primarily underscored the role of three major functional gene sets in ASD pathogenesis, namely (a) genes involved in neuronal communication, including synaptic function, (b) genes involved in epigenetic and gene expression regulation, and (c) genes involved in immune functions (De Rubeis et al., 2014; Satterstrom et al., 2020). Both GSEA and g:profiler analyses confirm in the present study the importance of the neuronal gene set. In particular, eight out of 10 gene sets selected by GSEA are implicated in “neurogenesis,” “neuron differentiation,” “neuron projection,” “synapse,” “synaptic signaling” and similar. Synaptic function regards all six cellular components identified by g: Profiler, as well as two biological processes, namely “negative regulation of synaptic transmission,” (GO: 0050805) and “modulation of excitatory postsynaptic potential” (GO:0098815)” (Table 5). As expected, gene sets are partly overlapping, with *MAPK8IP2* and *SHANK3* most present in different pathways. Pathway analysis network for autism already showed that the MAPK pathway is strongly linked to ASD and connected to other pathways (Wen et al., 2016). Also some commonly detected genes, like *CYFIP1*, share the same pathways as rare candidate genes like *DMD*, *SHANK3*, and *CHRNA7*. Interestingly, our study finds very few genes, if any, involved in transcriptional regulation and immune function (De Rubeis et al., 2014; Satterstrom et al., 2020). Whether this is due to interethnic differences in pathogenetic mechanisms or to a sampling bias due to a relatively small sample size assessed in the present study, will have to be determined.

The present study has several limitations. First, our sample is sizable, considering that it was recruited from a novel North African ethnic group, but also relatively small compared to large‐scale studies addressing ASD genetics. Hence negative results, like the absence of GO epigenetic and transcriptional regulation genes in GSEA, should be viewed with caution until replicated in a different sample from the same ethnic group, as they may reflect a sampling bias. Second, parents were not collected and analyzed, so we cannot define whether CNVs are de novo or inherited. This limitation is especially relevant considering that over 20% of parental pairs are consanguineous. Furthermore, this study is focused on aCGH and does not represent a broad‐spectrum genetic assessment. Small CNVs and single nucleotide mutations potentially responsible for the appearance of some core symptoms in our patients are not detected by aCGH technology. WES will thus be necessary to obtain a more complete picture of the genetic architecture of ASD in Tunisia (Srivastava et al., 2019). Finally, the contribution of gene–environment interactions to ASD etiology is receiving growing attention (Bai et al., 2019) and falls outside the scope of the present study. We have recently found altered plasma levels of some heavy metals and rare‐earth elements in a large subgroup including 89 children of this cohort, compared to a control group (Chehbani et al., 2020). The absorption, storage, management, transportation, and elimination of these elements from the body of these children could be altered and interact with genetic underpinnings in clinically meaningful ways (Bai et al., 2019).

### 4.1 Conclusion

To our knowledge, this is the first genetic investigation applying aCGH technology in a Tunisian sample of patients with ASD. Despite the limitations discussed above, our results support the use of aCGH as a first‐tier genetic test for ASD, both (a) to identify recurrent CNVs and genetic syndromes associated with ASD, and (b) to detect rare or “unique” pathogenic and likely pathogenic CNVs. This level of genetic characterization can, at least in some cases, foster a better understanding of phenotype–genotype correlations and improved management at the clinical level (Butler et al., 2022). At the same time, these results confirm that despite using the most advanced technologies, only a minority of cases with ASD finds a satisfactory monogenic explanation to their disorder. Our understanding of the genetic maze in ASD is still limited. Gene–environment interactions may represent the plausible explanation for CNVs currently with unknown clinical significance, as well as for cases of ASD due to genetics and epigenetics jointly deranging their prenatal and early postnatal neurodevelopment.

## CONFLICT OF INTEREST

The authors declare no conflict of interest.

## AUTHOR CONTRIBUTIONS

Fethia Chehbani, Naoufel Gaddour, Maria Luisa Scattoni, and Antonio M. Persico conceived the study and participated in study design; Fethia Chehbani, and Naoufel Gaddour collected the patients' history, accomplished the medical work‐up, collected blood samples, and performed psychological testing; Fethia Chehbani performed genomic DNA extraction; Chiara Picinelli, Marco Baccarin, and Paola Castronovo performed a‐CGH laboratory analysis; Pasquale Tomaiuolo, Fethia Chehbani, Marco Baccarin, and Antonio M. Persico analyzed and scored a‐CGH data; Fethia Chehbani wrote the manuscript; Pasquale Tomaiuolo, Maria Luisa Scattoni, and Antonio M. Persico revised the manuscript. All authors approved the final manuscript as submitted and agree to be accountable for all aspects of the work.

## ETHICS APPROVAL

This study was approved by the Ethics Committee of Mahdia's University Hospital, Tunisia (Ref. n. *PO9PSY‐2018*). The purpose and protocol of the study were explained to parents by the child psychiatrist (N.G.) and the first investigator (F.C.). Written informed consent was obtained from parents of all patients.

## Supporting information


Supinfo

Table S1

## Data Availability

The data that supports the findings of this study are available in the supplementary material of this article.
