# Supplementary material for: Yield of array‐CGH analysis in Tunisian children with autism spectrum disorder
Source: Mol Genet Genomic Med. 2022 Jun 27;10(8):e1939. doi: 10.1002/mgg3.1939 (PMC9356560; doi:10.1002/mgg3.1939)
Supplement: Supplementary file 1 — Supinfo [file MGG3-10-e1939-s002.docx]

**SUPPLEMENTARY MATERIAL**

**Yield of Array-CGH Analysis in Tunisian Children with**

**Autism Spectrum Disorder**

**Running title: Array-CGH for autism in Tunisia**

Fethia Chehbani^1,2*^, Pasquale Tomaiuolo^3^, Chiara Picinelli^3^, Marco Baccarin^3,4^, Paola Castronovo^3^, Maria Luisa Scattoni^5^, Naoufel Gaddour^6^, and Antonio M. Persico^7^

ˡDepartment of Psychiatry, Research Laboratory “Vulnerability to Psychotic Disorders LR 05 ES 10”, Monastir University Hospital, Monastir, Tunisia

²Faculty of Pharmacy, University of Monastir, Monastir, Tunisia

³Mafalda Luce Center for Pervasive Developmental Disorders, Milan, Italy

^4^Synlab Suisse SA, Department of Genetics, Bioggio / Switzerland

^5^Research Coordination and Support Service, Istituto Superiore di Sanità, Rome, Italy.

^6^Unit of Child Psychiatry, Monastir University Hospital, Monastir, Tunisia

^7^Child & Adolescent Neuropsychiatry Program, Modena University Hospital & Department of Biomedical, Metabolic and Neural Sciences, University of Modena and Reggio Emilia, Modena, Italy

**Corresponding Author**: Prof. Antonio Persico, Child & Adolescent Neuropsychiatry, University of Modena and Reggio Emilia, via Giuseppe Campi 287, I-41125 Modena, Italy; phone number +39-059-2055372; fax +39-059-2055625; email antonio.persico@unimore.it.

**Supplementary Table 1.** List of all common and rare CNVs found in 98 Tunisian patients with Autism Spectrum Disorder. All genes present in each CNV are listed. OMIM genes are followed by their OMIM id. n. Genes highlighted in bold and black are located in rare CNVs and are most responsible for pathogenicity score, whereas genes highlighted in bold and red are autism genes listed in the SFARI database and located in common CNVs.
